# The ‘Amoeboid Predator-Fungal Animal Virulence’ Hypothesis

**DOI:** 10.3390/jof5010010

**Published:** 2019-01-21

**Authors:** Arturo Casadevall, Man Shun Fu, Allan J. Guimaraes, Patricia Albuquerque

**Affiliations:** 1 Department of Molecular Microbiology and Immunology, Johns Hopkins School of Public Health, Baltimore, MD 21205, USA; sarahfu52@gmail.com; 2 Department of Microbiology and Parasitology, Biomedical Institute, Fluminense Federal University, Niterói, Rio de Janeiro 24020-141, Brazil; allanguimaraes@id.uff.br; 3 Faculty of Ceilandia, University of Brasilia, Brasilia DF 70904-970, Brazil; pattycherie@gmail.com

**Keywords:** fungi, amoeba, virulence, pathogenicity, *Cryptococcus*, *aspergillus*

## Abstract

The observation that some aspects of amoeba-fungal interactions resemble animal phagocytic cell-fungal interactions, together with the finding that amoeba passage can enhance the virulence of some pathogenic fungi, has stimulated interest in the amoeba as a model system for the study of fungal virulence. Amoeba provide a relatively easy and cheap model system where multiple variables can be controlled for the study of fungi-protozoal (amoeba) interactions. Consequently, there have been significant efforts to study fungal–amoeba interactions in the laboratory, which have already provided new insights into the origin of fungal virulence as well as suggested new avenues for experimentation. In this essay we review the available literature, which highlights the varied nature of amoeba-fungal interactions and suggests some unsolved questions that are potential areas for future investigation. Overall, results from multiple independent groups support the ‘amoeboid predator–fungal animal virulence hypothesis’, which posits that fungal cell predation by amoeba can select for traits that also function during animal infection to promote their survival and thus contribute to virulence.

## 1. Introduction

Fungi are major pathogens of plants, insects and ectothermic vertebrates but there are relatively few that are able to cause disease in mammals. The remarkable resistance of mammals to fungal diseases is attributed to a combination of endothermy, which creates a thermal restriction zone for survival of most fungal species and adaptive immunity [1,2,3]. However, that still leaves unresolved the question of how the capacity for virulence emerged in soil microbes that have no need for an animal host for survival or replication. This question is particularly relevant since for most pathogenic fungi there is no human-to-human transmission and thus it is unlikely that their virulence traits emerged from animal selection [4]. In 2001 a comparative analysis of the replication of *Cryptococcus neoformans* in macrophages and the free-living amoeba *Acanthamoeba castellani* revealed remarkable similarities leading to the proposal that its capacity for virulence emerged accidentally from selection by amoeboid predators in soils [5]. Subsequent work extended this concept to other pathogenic fungal species such as *Aspergillus* spp., *Histoplasma capsulatum*, *Blastomyces dermatitides*, *Sporothrix schenkii*, *Cryptococcus gattii* and entomogenous fungi [6,7,8,9]. Amoeba have putative mannose receptors that allow them to recognize this sugar in fungal surfaces [10,11].

In the past two decades, the concept that amoeba serve as selection mechanism leading to the emergence of virulence in different microbes has gained credence [12,13,14] and amoeba have emerged as a major model system for the study of the evolution of virulence in many types of microorganisms, including fungi [15]. The nature of amoeba, which includes food acquisition by phagocytosis and the fact that the fungi spend most of their life cycle in soil corroborates this hypothesis. Besides, macrophages and amoeba have similar mechanisms of phagocytosis and prey inactivation [16]. In fact, the connection between food acquisition by phagocytic cells and host defense may be ancient and it has been proposed that the digestive and immune systems of Metazoa share a common origin in deep time [17]. In this essay, we review the literature on interactions between fungal virulence and amoeba and discuss the state of the hypothesis that amoeboid predators select for the capacity of animal virulence in soil fungi. For readers interested in this subject, we note other recent reviews on the topic [18,19,20].

## 2. Early Studies on the Interaction of Fungi and Amoeba

The interaction of amoeba with animal pathogenic fungi has been studied for decades. In 1931, Castellani reported an amoeba growing in cultures of a *Cryptococcus* spp. [21], providing an early report of fungal–amoeba interactions whereby the amoeba appear to lyse the yeast cells. In 1955, *A. castellanii* was shown to feed on *C. neoformans* [22] and subsequently demonstrated to feed on cultures of *Torula famata* and *Candida parapsilosis* [23]. Interestingly, this early study reported that growth on the yeast *C. parapsilosis* was much faster than growth on the filamentous *T. famata* since the protozoa had difficulty ingesting hyphae [23]. Beginning in the late 1970s, Bulmer and colleagues carried out studies of the interaction of *C. neoformans* with amoeba and established that the protozoa preyed and devoured encapsulated cells with the emergence of hyphal cells as resistant forms, which were hypovirulent in mice [24,25]. Furthermore, this group with their colleagues reported that amoeba were biotic control factors for *C. neoformans* in the environment [26], establishing an ecological correlate of relevance for laboratory observations involving fungal and protozoal cells. In other studies, *A. castellanii* was shown to rapidly kill cultures of *S. cerevisiae*, reducing more than half of the fungal cells after 90 min of co-incubation [27].

The focus of this review is on animal pathogenic fungi with particular emphasis on those that cause disease in mammals, but we note that other investigators have also explored the interaction of soil amoeba with other groups of soil fungi, including yeast and filamentous fungi acting in nature as saprophytes, entomopathogens and even as phytopathogens [28,29,30,31,32]. Although the observations also reveal a great number of different outcomes for those interactions, in general yeast cells were more prone to be ingested and killed by soil amoeba in comparison to hyphae. Esser and colleagues reported that pigmented conidia of plant pathogens *Alternaria*, *Curvularia*, *Helminthosporium* were internalized by *Thecamoeba* spp. and later egested in a viable condition, in a closer resemblance of nonlytic exocytosis [29]. Despite that, there are groups of amoebae, such as giant amoebae from the Vampyrellid family that have developed efficient strategies to attack and kill fungal spores by perforation [31]. The early studies established that amoeba could prey on animal pathogenic fungi, that there were differences in the fungal resistance to amoeba predation, and that different morphological forms of certain fungal species manifested differences in their resistance to protozoal ingestion.

## 3. Correspondence of Virulence Factors for Animals and Amoeba

### 3.1. C. neoformans

**Figure 1:**
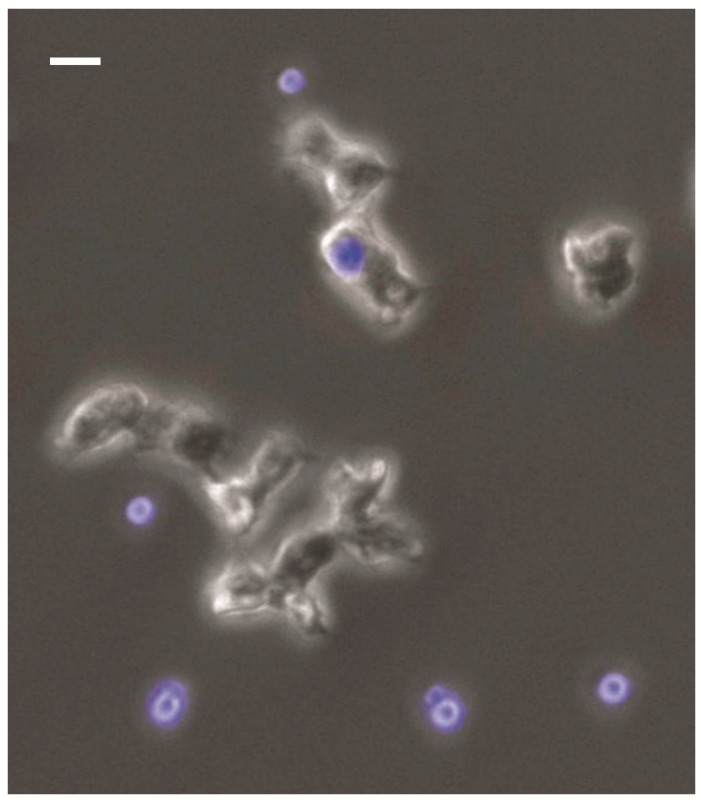
Interaction of *C. neoformans* (blue) with *Acanthamoeba castellanii*. The amoeba are much larger than the fungal cells and readily ingest them. The amoeba in the top middle center has ingested a fungal cell. For details of the conditions in this experiment see [45]. Briefly, *A. castellanii* and *C. neoformans* cells were incubated in a 8-well-chambered cover glass. Fungal cells appear blue because theys were stained with 0.01% Uvitex 2B prior to co-incubation. Images were taken using a DAPI (4′,6-diamidino-2-phenylindole) filter-equipped Zeiss Axiovert 200M inverted microscope with 20× phase objective.

**Figure 2:**
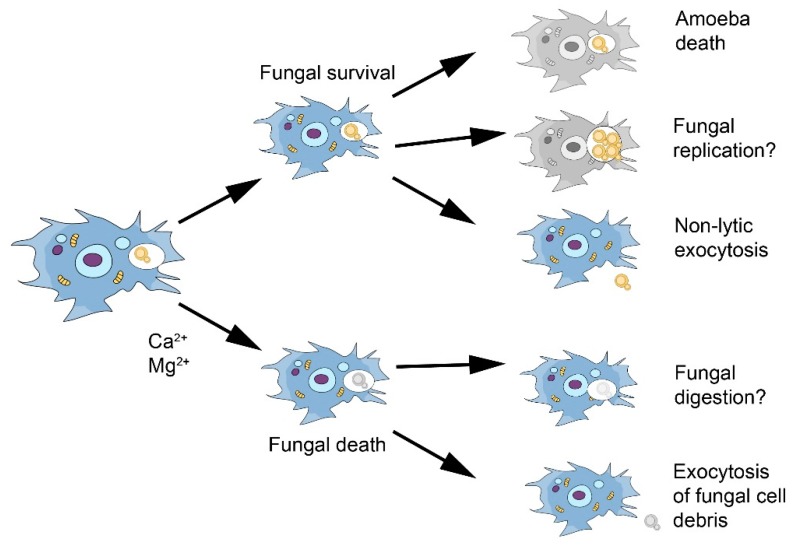
Scheme of known and possible interactions of *C. neoformans* with amoeba based on the experience with *Acanthamoeba castellanii*. The outcome of the interaction is highly variable and determined by such variables as the nutritional state of amoeba and the presence of metal cations in the media [45,46]. Question marks are added to processes for which there is uncertainty and/or that have not been demonstrated experimentally.

Among the animal pathogenic fungus *C. neoformans* is the most extensively studied species with regards to interactions with protozoa (Figure 1 and Figure 2). *C. neoformans* has several well defined virulence factors for animals that have been evaluated for their need in fungal survival against amoeba predation. The capsule, melanin synthesis and phospholipase have each been shown to be important for *C. neoformans* to resist predation by *A. castellanii* [5]. These virulence factors appear to have similar functions in protecting *C. neoformans* against both host defense mechanisms and amoeba predation. For example, the polysaccharide capsule interferes with phagocytosis by both macrophage and *A. castellanii* [33]. However, not every virulence determinant is relevant in both animals and protozoa. *C. neoformans* alpha mating locus strains are more virulent in mice than congenic ‘a’ mating locus strains but there was no difference in the survival of fungal cells of different mating types when confronted by amoeba [34]. Similarly, urease has been shown to be important for *C. neoformans* virulence in mice [35] but comparison of urease positive and deficient strains revealed no difference for survival with amoeba [36]. Mannitol production by *C. neoformans* is associated with pleiotropic effects on virulence but how this relates to its role in the environment is unknown [37,38,39]. The absence of one-to-one correspondence with regards to virulence factor importance in protozoa and *Animalia* is not surprising given that the phenomenon of virulence in metazoan hosts is much more complicated by the existence of organ systems and immune systems that can impart host damage when they respond to infection. For example, the major role for urease in the environment appears to be nutrient acquisition, but in the mammalian host this enzyme affects brain invasion [40,41], as well as the macrophage response to infection by virtue of its ability to alkaline the local environment through the generation of ammonia [36]. There is also increasing evidence that host damage in animal cryptococcosis is the result of immune-mediated damage [42,43,44], which implies that antigens irrelevant to surviving in the environment could contribute to virulence simply by eliciting malevolent immune responses.

In addition to the correspondence between animal and amoeba survival for selected fungal cells expressing certain virulence factors, there are also remarkable similarities in several cellular processes. *C. neoformans* responds to both amoeba and macrophages by enlarging its polysaccharide capsule, in what appears to be a protective stress response [47]. The capsular response is triggered by phospholipids that are presumably released from amoeba and macrophages by the action of cryptococcal enzymes, including phospholipase B [47]. The process for this mechanism envisions the release of enzymes by *C. neoformans* into its immediate environment that damage macrophage or protozoal cell membranes, producing polar phospholipids that are sensed by the fungal cell [47]. Hence, enzyme release followed by phospholipid sensing may be an early warning system in the environment that alerts the fungal cell that an amoeboid predator is in the environment. The occurrence of a similar process during infection would explain why residence in any particular host is associated with capsular enlargement, which in turn would protect against macrophage phagocytosis and killing. Incidentally, the process of phospholipid-mediated capsular enlargement can trigger the phenomena of giant (Titan) cell formation, with the emergence of enormous fungal cells [47], incapable of being engulfed. The phenomenon of *C. neoformans* non-lytic exocytosis (vomocytosis), a process discovered in macrophages [48,49], was subsequently observed to also occur with *A. castellanii* [50] and *D. discoideum* [51], providing another parallel between mammalian and protozoal cells that could represents an escape mechanism to exit predatory cells. At the cellular level exocytosis from both macrophages and slime mold cells have been shown to rely on actin polymerization driven by similar cellular machinery [52]. Another interesting parallel involves the release of fungal extracellular vesicles [53], which have been shown to affect macrophage function [54] and to interfere with amoeba metabolism [55]. *C. neoformans* cells produce 3-hydroxy fatty acids, which inhibit amoeba phagocytosis and could have a similar role during infection for macrophages [56].

More direct evidence for the ability of amoeba to affect *C. neoformans* virulence comes from passage experiments of an attenuated cryptococcal strain with *D. discoideum*, which showed a rapid increase in virulence [57]. This work also illustrated how the phenomenon of increased microbial virulence in setting of impaired hosts applied at the cellular level, since a *D. discoideum* mutant defective in myosin VII synthesis was susceptible to a highly attenuated acapsular *C. neoformans* strain [57]. Another protozoa that can prey on *C. neoformans* are *Paramecium* spp, which readily ingested and killed cryptococcal cells [58].

### 3.2. Aspergillus spp.

Like *C. neoformans*, there is circumstantial evidence that amoeba and *Aspergillus* spp. interact in the environment. Analysis of the environmental microflora in moisture-damaged buildings revealed the co-habitation of molds and amoeba [59]. Laboratory studies of amoeba and molds isolated from moisture-damaged buildings suggested that *Aspergillus* spp. benefitted from the presence of the protozoa while amoeba were indifferent to the presence of molds [60]. In parallel to the experience with *C. neoformans*, the interaction of *Aspergillus fumigatus* conidia with *A. castellanii* was found to have remarkable similarities to their interactions with avian macrophages [8]. In addition to that, the interaction of *A. fumigatus* with another amoeba, *Vermamoeba (Hartmannella) vermiformis*, promoted fungal filamentation and growth of and the major reduction of amoeba viability, while in the same conditions the interaction of this fungus with *A. castellanii* had no influence in fungal growth [61]. The authors also found that amoeba supernatants were able to increase germination and fungal growth. According to them the release of amoeba metabolites that can be used as nutrients by fungal cells might contribute to fungal persistence. Analysis of the interaction of *A. fumigatus* with the social amoeba *D. discoideum* revealed concordance between virulence factors for animals and those needed for fungal survival and killing of amoeba [62]. Specifically, gliotoxin was found to be important for killing *D. discoideum* thus linking the production of mycotoxins to predator control and fungal environmental survival [62]. Another mycotoxin fumagillin was shown to inhibit the growth of *Entamoeba histolytica* [63], and on the other hand, contribute to epithelial cell damage by allowing fungal invasion [64]. Substances from spores of *Aspergillus* spp. have been shown to have toxic effects on *Naegleria gruberi* and to mediate anti-phagocytic effects on human neutrophils and macrophages [65,66]. *Aspergillus* spp. DHN melanin, which inhibits phagolysosomal acidification also in macrophages interferes with phagocytosis by amoeba, suggesting that this virulence determinant has different functions for fungal cell survival with confronted by animal and protozoal phagocytic cells [67].

### 3.3. Candida spp.

Relatively little work has been done to explore amoeba-*Candida* spp. interactions. Although commensal *Candida* spp. limited to mammalian hosts would not be expected to be under predation by ameba in soils, there are some body sites that are co-inhabited by fungal and protozoal cells where they could potentially interact. For example, amoeba are present in the human oral cavity [68] where *Candida* spp. are also commonly found in a commensal state. Steenbergen et al. reported that *A. castellanii* preyed on *C. albicans* and efficiently reduced colony-forming units consistent with fungal killing [5]. In another work, the free soil amoeba *V. vermiformis* was shown to be able to internalize yeast cells of *C. albicans*, *C. glabrata* and *C. parapsilosis* and promote their proliferation in tap water with or without saliva traces [69]. Recently the interaction of *Candida* spp. and *S. cerevisiae* with *D. discoideum* and other non-axenic social amoeba was studied, including mutant strains of both the fungal and protozoal spp. [70]. This study reported that the outcome of the fungal–amoeba interaction could be altered by mutations in either party providing a system for the studying the effect of fungal and amoeba genes in determining which eukaryotic cells maintain ascendancy in the confrontation [70].

### 3.4. Other Pathogenic Fungi

**Figure 3:**
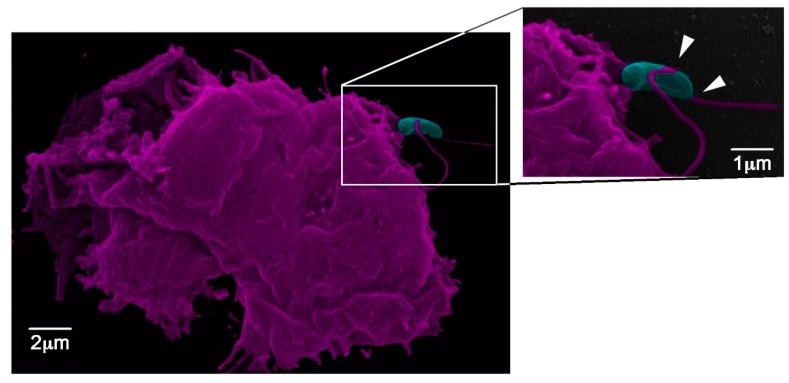
*Acanthamoeba castellanii* interaction with yeasts of *Sporothrix brasiliensis*. The white arrows depict the acanthapodes as projections from the *A. castellanii* surface in close contact with the fungal cell wall outer layer.

One study has investigated the interaction of *B. dermatitides*, *H. capsulatum* and *S. schenkii* (Figure 3) with *A. castellanii* and reported that each of these fungal species was able to grow in the presence of the amoeba [6]. The interaction of *Fusarium* spp. with amoeba is of interest given that both are involved in corneal infections. Amoeba have been proposed to be major biotic control factors for *Fusarium oxysporum* in soils [71]. Given that amoeba could be important contaminants of contact lens cleaning solutions, there could be situations when these two organisms interact in vivo [72,73]. Incubation of *Fusarium* with two different types of amoeba, *A. castellanii* and *V. vermiformis*, showed that although fungal cells were ingested the interaction resulted in enhanced fungal growth [74]. Moreover, there is evidence that the interaction of *Fusarium* spp. with *A. castellanii* can enhance the virulence of both organisms [73]. Indeed, severe keratitis caused by dual infection with *Fusarium* spp. and *A. castellanii* has been reported with the suggestion that this condition should be suspected in situations of refractory infection [75].

## 4. Amoeba and Fungal Dimorphism

An emerging theme in amoeba-fungal studies is that hyphal forms are more resistant to protozoal predation than yeast forms. This was noted as early as 1964 when Nero et al. reported that a mold was more resistant to amoeba than *Candida parapsilosis* [23]. Similarly, Bulmer et al. noted amoeba predation of *C. neoformans* lawns resulted in the emergence of pseudohyphal forms, which were resistant to amoeba but less virulent in mice [25]. Analysis of pseudohyphal strains showed a hypermutation locus in the RAM signaling pathway [76], suggesting that emergence of such forms in experiments involving amoeba predation could reflect mutations in this pathway. Incubation of *B. dermatitides*, *H. capsulatum* and *S. schenkii* yeast cells with *A. castellani* at 37 °C led to the rapid emergence of filamentous forms for each fungal species even at a temperature where the yeast form is preferred [6]. Recently, incubation of *A. fumigatus* conidia with the free living amoeba *V. vermiformis* was reported to promote fungal filamentation and growth [61]. *C. albicans* forms hyphae inside or outside *D. discoideum*, but no hyphae formation without amoeba [70]. Comparison of phagocytosis of *C. neoformans* hyphae, pseudohyphae and yeast forms showed that the elongated forms were resistant to phagocytosis by both macrophages and amoeba [77]. However, *C. neoformans* cells with hyphal and pseudohyphal morphology were much less virulent in mice and moths, indicating that resistance to one host may be associated with vulnerability in another [76]. The differences in susceptibility between yeast and hyphal forms raises the question of whether amoeba predation has been a contributing factor in the evolutionary emergence of fungal dimorphism. In this regard, the emergence of hyphal forms of *C. neoformans* during amoeba predation of cryptococcal lawns in agar was considered an ‘escape hatch’ for the survival of some cells [78].

## 5. Considerations, Caveats and Unsolved Questions

### 5.1. Insights Drawn Primarily from a Few Amoeba and Fungal Species

Most of the studies of fungal–amoeba interactions have been conducted with *A. castellanii* grown in axenic media. Amoeba that have been selected in laboratory conditions to grow in axenic conditions may exhibit very different physiology than wild amoeba. In general, amoeba recovered from the environment do not grow in culture conditions and thus are difficult to use for fungal–amoeba interaction studies. Given the tremendous variety of amoeba in the environment, the reliance on *A. castellanii* in laboratory studies raises the concern that insights gained with this protozoal species may not be representative of most fungal–amoeba interactions in nature. Similarly, the relatively few studies of fungal interactions with *D. discoideum* have also been done with laboratory strains. Hence, the limitations of current systems and the paucity of amoeba strains that have been examined suggest caution in extrapolating laboratory results from environmental conditions. One potential resource to overcome this problem could be the studies of fungal pathogens with mycophagous amoeba. Both *A. castellanii* and *D. discoideum* have been shown to be more adapted to have bacterial cells as food sources. Although we can find a few studies about the interaction of amoeba species that in nature feed primarily on fungal cells, there is a general lack of knowledge of the interaction of mycophagous amoeba with animal fungal pathogens [79,80]. One line of fungal–amoeba interaction still neglected is the potential of amoeba working as Trojan horses for animal fungal infections, as may occur with other intracellular pathogens. Considering the number of fungal species that are able to not only survive but also replicate inside soil amoeba, there is the possibility of these soil organisms work as vectors for some fungal infections.

### 5.2. Ascendancy in Fungal–Amoeba Interactions

Which entity prevails in fungal–amoeba confrontations is highly dependent on the experimental parameters. As evident from the review of the available literature, fungi prevailed in some studies while amoeba were ascendant in others. Clearly, there are major differences in the ability of individual fungal species to resist amoeba predation, a finding that dates to early studies [23]. In this regard, *C. neoformans* and other pathogenic fungi with environmental habitats were reported to be more resistant to *A. castellanii* than *C. albicans* or laboratory adapted *S. cerevisiae* strains [5,6]. There are also differences between amoeba species in their predatory capacity. Hence, who wins in fungal–amoeba confrontations is highly dependent on the identity of the players involved. There is also evidence that the experimental conditions can benefit either the fungi or amoeba. For example, confrontations of *C. neoformans* with *A. castellanii* in phosphate buffered saline result in fungal growth while confrontations in amoeba media result in fungal predation with reduction of cryptococcal colony-forming units [46]. Although this result was initially interpreted as implying that amoeba in a better nutritional state would be stronger predators for *C. neoformans*, recent findings suggest that the enhanced protozoal activity could have resulted from divalent cations amoeba media. In this regard, the presence of Ca^2+^ and Mg^2+^ have strong effects in potentiating *A. castellanii* antifungal activity [45], suggesting that differences in soil cation concentrations could affect the relative predatory activity abundance of fungi and amoeba.

### 5.3. Animal Pathogenic Fungi–Amoeba Interactions in Context

Although from our anthropomorphic vantage point we tend to focus on animal and human pathogenic fungi, it is important to note that this is a minute part of fungal interactions with other hosts ranging from those with Protista, Plantae and Animalia. Indirect evidence for the longstanding nature of amoeba-fungal interactions comes from the finding that higher fungi synthesize lectins that are toxic to amoeba, a finding consistent with the development of inducible fungal anti-protozoal defense mechanism [81]. There is a large number of fungal species that parasitize amoeba known as amoebophagous fungi, which are poorly understood because it is difficult to study them outside of the parasitic lifestyle [82]. The impact of such amoebophagous fungi on Protista evolution and their consequences for interactions of amoeba with other fungi are unclear. Similarly, interactions of amoeba with the mycorrhizosphere represent very different ecological spaces for the selection of amoeba and fungal traits to survive their interactions [83]. The point here is that conclusions drawn from observations made in controlled interactions of fungi and amoeba in the laboratory need to acknowledge the limitations inherent in their simplicity relative to vast complexity of protozoal–fungal interactions in the biosphere.

### 5.4. Fungal–Amoeba Interaction and Susceptibility to Antifungals

Due to the enormous genetic variety of the community of microorganisms in the soil, each possesses a unique combination of characteristics and distinct attributes to avoid the action of phagocytic predators, such as chemical defenses, including the expression of capsule in *Cryptococcus* sp. and mechanisms for iron acquisition and biofilm formation in *Candida* spp. Once adapted to the intracellular environment of amoebas, these pathogens could also be protected from the action of biocides and environmentally harsh conditions, making their survival and dissemination more effective. Therefore, the expression of these fungal virulence traits should be also considered in the extent to which amoeba predation could influence susceptibility to antifungal drugs. In this regard, passage of fungi from sites inhabited by birds in amoeba reduced their susceptibility to amphotericin B [84]. Although the mechanism for this effect is uncertain it is possible that adaptation to amoeba is associated with metabolic changes that reduce susceptibility to polyenes, highlighting how tangential interactions in the environment can reverberate into findings of medical importance. For example, the antibody binding to the capsule of *C. neoformans* triggers transcriptional changes to lipid genes that affect the susceptibility to antifungal agents [85]. In a similar vein, the reduced susceptibility to amphotericin b following interactions with amoeba can be an incidental result of a new metabolic state triggered by protozoa-fungal interactions.

## 6. A Restatement of the Amoeboid Predator—Animal Virulence Hypothesis

The idea that animal pathogenic fungi acquired many of their characteristics for virulence from interactions with soil protozoal is almost two decades old [5]. The origin of virulence for the entomopathogenic fungi *Metarhizium anisopliae* and *Beauveria bassiana* has also been proposed to involve interactions with soil amoeba selected for traits that allow survival in insect hemophytes, which are host phagocytic defensive cells [9]. Despite its increasing acceptance and application to other pathogenic fungi, this hypothesis has not been named. Here we propose to call the process the ‘amoeboid predator-animal virulence’ hypothesis in a phrase that captures the basics of this idea.

**Figure 4:**
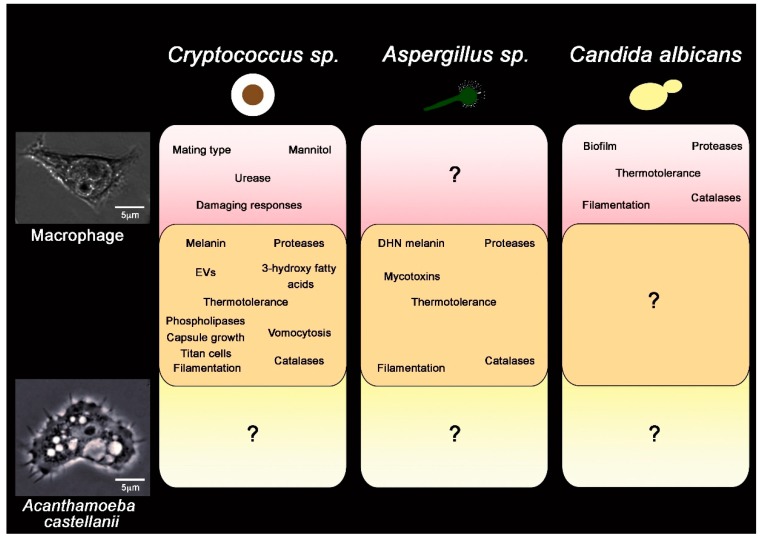
Summary of described virulence factors for three major fungal pathogens, in the context of their importance in the interaction with macrophage (pink), amoeba (yellow) or both (orange). For both *Cryptococcus* and *Aspergillus* spp. Several attributes have described that are important for both fungal cell survival in both amoeba and macrophages (orange box, center). For *C. albicans* comparable studies in amoeba have not been undertaken. Question marks denote uncertainty about traits specific to either macrophages or amoeba.

The amoeboid predator-animal virulence hypothesis posits that constant amoeboid predation over eons selected for fungal traits that also facilitate survival in certain animal hosts and thus confer on those fungal species the capacity for virulence. According to this view such virulence factors in *C. neoformans* as the polysaccharide capsule, melanin synthesis and phospholipase production have protective roles in the environment that are equally adapted to protection against macrophages during animal infection [5] (Figure 4). For *Aspergillus* spp., melanin and mycotoxins tripacidin, gliotoxin and fumagillin serve similar functions during its interaction with amoeba and macrophages [18]. Although one cannot expect on-to-one correspondence in function for virulence factors with host cells that diverged eons ago what is most salient is the overall similarities for the survival and cytotoxic processes involved in these fungal cell–amoeba interactions.

A central feature of the amoeboid predator-animal virulence hypothesis is that it posits that the capacity for virulence can emerge independent of the final host. The phenomenon was termed ‘accidental virulence’ in prior essays [86]. The notion that interactions with amoeba select for traits that enhance resistance against host defense cells has now been extended to several pathogenic microbes including mycobacteria [87] and numerous other microorganisms [12,20,88,89]. One question that emerges from the amoeboid predator-animal virulence hypothesis is why only a small minority of soil microbes have the capacity for animal virulence when all are presumably being selected by amoeboid predators. One possible answer is that the capacity for virulence is a complex phenotype that requires more traits than are needed for survival in soils and, as such, is found only in a rare microbial species. This view was put forth as a metaphorical situation where microbes each had some ‘cards’ that in certain combinations conferred the capacity for virulence in some situations but not others [90]. For example, the ability to cause disease in warm-blooded hosts would require thermotolerance, the capacity to survive at higher temperatures. Another explanation could include the notion that among the soil survival strategies available, some are more suitable for conferring the capacity of virulence for animal hosts. In this regard a comparison of the anti-phagocytic ability of *Cryptococcus* spp., each of which have capsules, found that the capsule of *C. neoformans* was the most effective in protecting fungal cells against macrophages [91]. Alternatively, for some microbes the strategies used for defense against environmental predators may be totally different from those needed to survive in animal hosts, which may cause a fitness trade-off and result in a decrease in pathogenicity. We also need to consider that mammals comprise only a small fraction of any environmental niche and that a focus on traits that promote survival in mammals could miss many other attributes of virulence that were selected by fungal interaction with soil predators that are more suitable to interact with many other potential hosts.

## References

[1] Robert V.A., Casadevall A. (2009). Vertebrate endothermy restricts most fungi as potential pathogens. J. Infect. Dis..

[2] Bergman A., Casadevall A. (2010). Mammalian endothermy optimally restricts fungi and metabolic costs. mBio.

[3] Casadevall A. (2012). Fungi and the rise of mammals. PLoS Pathog..

[4] Desjardins C.A., Giamberardino C., Sykes S.M., Yu C.H., Tenor J.L., Chen Y., Yang T., Jones A.M., Sun S., Haverkamp M.R. (2017). Population genomics and the evolution of virulence in the fungal pathogen *Cryptococcus neoformans*. Genome Res..

[5] Steenbergen J.N., Shuman H.A., Casadevall A. (2001). *Cryptococcus neoformans* interactions with amoebae suggest an explanation for its virulence and intracellular pathogenic strategy in macrophages. Proc. Natl. Acad. Sci. USA.

[6] Steenbergen J.N., Nosanchuk J.D., Malliaris S.D., Casadevall A. (2004). Interaction of *Blastomyces dermatitidis*, *Sporothrix schenckii*, and *Histoplasma capsulatum* with *Acanthamoeba castellanii*. Infect. Immun..

[7] Malliaris S.D., Steenbergen J.N., Casadevall A. (2004). *Cryptococcus neoformans* var. *gattii* can exploit *Acanthamoeba castellanii* for growth. Med. Mycol..

[8] Van Waeyenberghe L., Bare J., Pasmans F., Claeys M., Bert W., Haesebrouck F., Houf K., Martel A. (2013). Interaction of Aspergillus fumigatus conidia with Acanthamoeba castellanii parallels macrophage-fungus interactions. Environ. Microbiol. Rep..

[9] Bidochka M.J., Clark D.C., Lewis M.W., Keyhani N.O. (2010). Could insect phagocytic avoidance by entomogenous fungi have evolved via selection against soil amoeboid predators?. Microbiology.

[10] Allen P.G., Dawidowicz E.A. (1990). Phagocytosis in Acanthamoeba: I. A mannose receptor is responsible for the binding and phagocytosis of yeast. J. Cell. Physiol..

[11] Allen P.G., Dawidowicz E.A. (1990). Phagocytosis in Acanthamoeba: II. Soluble and insoluble mannose-rich ligands stimulate phosphoinositide metabolism. J. Cell. Physiol..

[12] Molmeret M., Horn M., Wagner M., Santic M., Abu K.Y. (2005). Amoebae as training grounds for intracellular bacterial pathogens. Appl. Environ. Microbiol..

[13] Harb O.S., Gao L.Y., Abu Kwaik Y. (2000). From protozoa to mammalian cells: A new paradigm in the life cycle of intracellular bacterial pathogens. Environ. Microbiol..

[14] Hilbi H., Weber S.S., Ragaz C., Nyfeler Y., Urwyler S. (2007). Environmental predators as models for bacterial pathogenesis. Environ. Microbiol..

[15] Mylonakis E., Casadevall A., Ausubel F.M. (2007). Exploiting amoeboid and non-vertebrate animal model systems to study the virulence of human pathogenic fungi. PLoS Pathog..

[16] Davies B., Chattings L.S., Edwards S.W. (1991). Superoxide generation during phagocytosis by Acanthamoeba castellanii: Similarities to the respiratory burst of immune phagocytes. Microbiology.

[17] Broderick N.A. (2015). A common origin for immunity and digestion. Front. Immunol..

[18] Novohradska S., Ferling I., Hillmann F. (2017). Exploring Virulence Determinants of Filamentous Fungal Pathogens through Interactions with Soil Amoebae. Front. Cell. Infect. Microbiol..

[19] Balczun C., Scheid P.L. (2017). Free-Living Amoebae as Hosts for and Vectors of Intracellular Microorganisms with Public Health Significance. Viruses.

[20] Guimaraes A.J., Gomes K.X., Cortines J.R., Peralta J.M., Peralta R.H. (2016). Acanthamoeba spp. as a universal host for pathogenic microorganisms: One bridge from environment to host virulence. Microbiol. Res..

[21] Castellani A. (1931). An amoeba growing in cultures of a yeast. J. Trop. Med. Hyg..

[22] Castellani A. (1955). Phagocytic and destructive action of *Hartmanella castellanii* (Amoeba castellanii) on pathogenic encapsulated yeast-like fungus *Torulopsis neoformans* (*Cryptococcus neoformans*). Ann. Inst. Pasteur.

[23] Nero L.C., Tarver M.G., Hedrick L.R. (1964). Growth of *Acanthomoeba castellani* with the yeast *Torulopsis famata*. J. Bacteriol..

[24] Bunting L.A., Neilson J.B., Bulmer G.S. (1979). *Cryptococcus neoformans*: Gastronomic delight of a soil ameba. Sabouraudia.

[25] Neilson J.B., Fromtling R.A., Bulmer G.S. (1981). Pseudohyphal forms of *Cryptococcus neoformans*: Decreased survival in vivo. Mycopathologia.

[26] Ruiz A., Neilson J.B., Bulmer G.S. (1982). Control of *Cryptococcus neoformans* in nature by biotic factors. Sabouraudia.

[27] Bowen I.D.C., Coakley W.T., James C.J. (1979). The digestion of *Saccharomyces cerevisiase* by *Acanthamoeba castellanii*. Protoplasma.

[28] Heal O. (1963). Soil fungi as food for amoebae. Soil Organisms.

[29] Esser R., Ridings W., Sobers E. (1975). Ingestion of fungus spores by protozoa. Proc. Soil Crop Sci. Soc. Fla..

[30] Chakraborty S., Old K., Warcup J. (1983). Amoebae from a take-all suppressive soil which feed on Gaeumannomyces graminis tritici and other soil fungi. Soil Biol. Biochem..

[31] Old K., Darbyshire J. (1978). Soil fungi as food for giant amoebae. Soil Biol. Biochem..

[32] Old K. (1978). Perforation and lysis of fungal spores by soil amoebae. Ann. Appl. Biol..

[33] Zaragoza O., Chrisman C.J., Castelli M.V., Frases S., Cuenca-Estrella M., Rodriguez-Tudela J.L., Casadevall A. (2008). Capsule enlargement in *Cryptococcus neoformans* confers resistance to oxidative stress suggesting a mechanism for intracellular survival. Cell. Microbiol..

[34] Nielsen K., Cox G.M., Litvintseva A.P., Mylonakis E., Malliaris S.D., Benjamin D.K., Giles S.S., Mitchell T.G., Casadevall A., Perfect J.R. (2005). *Cryptococcus neoformans* {alpha} strains preferentially disseminate to the central nervous system during coinfection. Infect. Immun..

[35] Cox G.M., Mukherjee J., Cole G.T., Casadevall A., Perfect J.R. (2000). Urease as a virulence factor in experimental cryptococcosis. Infect. Immun..

[36] Fu M.S., Coelho C., De Leon-Rodriguez C.M., Rossi D.C.P., Camacho E., Jung E.H., Kulkarni M., Casadevall A. (2018). *Cryptococcus neoformans* urease affects the outcome of intracellular pathogenesis by modulating phagolysosomal pH. PLoS Pathog..

[37] Guimaraes A.J., Frases S., Cordero R.J., Nimrichter L., Casadevall A., Nosanchuk J.D. (2010). *Cryptococcus neoformans* responds to mannitol by increasing capsule size in vitro and in vivo. Cell. Microbiol..

[38] Chatuverdi V., Flynn T., Niehaus W.G., Wong B. (1996). Stress tolerance and pathogenic potential of a mannitol mutant of *Cryptococcus neoformans*. Microbiology.

[39] Hamilton A.J., Holdom M.D. (1999). Antioxidant systems in the pathogenic fungi of man and their role in virulence. Med. Mycol..

[40] Olszewski M.A., Noverr M.C., Chen G.H., Toews G.B., Cox G.M., Perfect J.R., Huffnagle G.B. (2004). Urease expression by *Cryptococcus neoformans* promotes microvascular sequestration, thereby enhancing central nervous system invasion. Am. J. Pathol..

[41] Shi M., Li S.S., Zheng C., Kim K.S., Zhou H., Kubes P., Mody C.H. (2010). Real-time imaging of trapping and urease-dependent transmigration of *Cryptococcus* in the brain. J. Clin. Investig..

[42] Neal L.M., Xing E., Xu J., Kolbe J.L., Osterholzer J.J., Segal B.M., Williamson P.R., Olszewski M.A. (2017). CD4(+) T Cells Orchestrate Lethal Immune Pathology despite Fungal Clearance during *Cryptococcus neoformans* Meningoencephalitis. mBio.

[43] Pirofski L.A., Casadevall A. (2017). Immune-Mediated Damage Completes the Parabola: *Cryptococcus neoformans* Pathogenesis Can Reflect the Outcome of a Weak or Strong Immune Response. mBio.

[44] Panackal A.A., Williamson K.C., van de Beek D., Boulware D.R., Williamson P.R. (2016). Fighting the Monster: Applying the Host Damage Framework to Human Central Nervous System Infections. mBio.

[45] Fu M.S., Casadevall A. (2017). Divalent metal cations potentiate the predatory capacity of amoeba for *Cryptococcus neoformans*. Appl. Environ. Microbiol..

[46] Garcia-Solache M.A., Izquierdo-Garcia D., Smith C., Bergman A., Casadevall A. (2013). Fungal virulence in a lepidopteran model is an emergent property with deterministic features. mBio.

[47] Chrisman C.J., Albuquerque P., Guimaraes A.J., Nieves E., Casadevall A. (2011). Phospholipids trigger *Cryptococcus neoformans* capsule enlargement during interactions with amoebae and macrophages. PLoS Pathog..

[48] Ma H., Croudace J.E., Lammas D.A., May R.C. (2006). Expulsion of live pathogenic yeast by macrophages. Curr. Biol..

[49] Alvarez M., Casadevall A. (2007). Cell-to-cell spread and massive vacuole formation after *Cryptococcus neoformans* infection of murine macrophages. BMC Immunol..

[50] Chrisman C.J., Alvarez M., Casadevall A. (2010). Phagocytosis of *Cryptococcus neoformans* by, and nonlytic exocytosis from, *Acanthamoeba castellanii*. Appl. Environ. Microbiol..

[51] Watkins R.A., Andrews A., Wynn C., Barisch C., King J.S., Johnston S.A. (2018). *Cryptococcus neoformans* Escape From Dictyostelium Amoeba by Both WASH-Mediated Constitutive Exocytosis and Vomocytosis. Front. Cell. Infect. Microbiol..

[52] Carnell M., Zech T., Calaminus S.D., Ura S., Hagedorn M., Johnston S.A., May R.C., Soldati T., Machesky L.M., Insall R.H. (2011). Actin polymerization driven by WASH causes V-ATPase retrieval and vesicle neutralization before exocytosis. J. Cell Biol..

[53] Rodrigues M.L., Nimrichter L., Oliveira D.L., Frases S., Miranda K., Zaragoza O., Alvarez M., Nakouzi A., Feldmesser M., Casadevall A. (2007). Vesicular polysaccharide export in *Cryptococcus neoformans* is a eukaryotic solution to the problem of fungal trans-cell wall transport. Eukaryot. Cell.

[54] Oliveira D.L., Freire-de-Lima C.G., Nosanchuk J.D., Casadevall A., Rodrigues M.L., Nimrichter L. (2010). Extracellular vesicles from *Cryptococcus neoformans* modulate macrophage functions. Infect. Immun..

[55] Rizzo J., Albuquerque P.C., Wolf J.M., Nascimento R., Pereira M.D., Nosanchuk J.D., Rodrigues M.L. (2017). Analysis of multiple components involved in the interaction between *Cryptococcus neoformans* and *Acanthamoeba castellanii*. Fungal Biol..

[56] Madu U.L., Ogundeji A.O., Pohl C.H., Albertyn J., Sebolai O.M. (2017). Elucidation of the Role of 3-Hydroxy Fatty Acids in *Cryptococcus*-amoeba Interactions. Front. Microbiol..

[57] Steenbergen J.N., Nosanchuk J.D., Malliaris S.D., Casadevall A. (2003). *Cryptococcus neoformans* virulence is enhanced after intracellular growth in the genetically malleable host *Dictyostelium discoideum*. Infect. Immun..

[58] Frager S.Z., Chrisman C.J., Shakked R., Casadevall A. (2010). Paramecium species ingest and kill the cells of the human pathogenic fungus *Cryptococcus neoformans*. Med. Mycol..

[59] Yli-Pirila T., Kusnetsov J., Haatainen S., Hanninen M., Jalava P., Reiman M., Seuri M., Hirvonen M.R., Nevalainen A. (2004). Amoebae and other protozoa in material samples from moisture-damaged buildings. Environ. Res..

[60] Yli-Pirila T., Kusnetsov J., Hirvonen M.R., Seuri M., Nevalainen A. (2006). Effects of amoebae on the growth of microbes isolated from moisture-damaged buildings. Can. J. Microbiol..

[61] Maisonneuve E., Cateau E., Kaaki S., Rodier M.H. (2016). *Vermamoeba vermiformis-Aspergillus fumigatus* relationships and comparison with other phagocytic cells. Parasitol. Res..

[62] Hillmann F., Novohradska S., Mattern D.J., Forberger T., Heinekamp T., Westermann M., Winckler T., Brakhage A.A. (2015). Virulence determinants of the human pathogenic fungus *Aspergillus fumigatus* protect against soil amoeba predation. Environ. Microbiol..

[63] Arico-Muendel C., Centrella P.A., Contonio B.D., Morgan B.A., O’Donovan G., Paradise C.L., Skinner S.R., Sluboski B., Svendsen J.L., White K.F. (2009). Antiparasitic activities of novel, orally available fumagillin analogs. Bioorgan. Med. Chem. Lett..

[64] Guruceaga X., Ezpeleta G., Mayayo E., Sueiro-Olivares M., Abad-Diaz-De-Cerio A., Aguirre Urizar J.M., Liu H.G., Wiemann P., Bok J.W., Filler S.G. (2018). A possible role for fumagillin in cellular damage during host infection by *Aspergillus fumigatus*. Virulence.

[65] Hobson R.P. (2000). The effects of diffusates from the spores of *Aspergillus fumigatus* and *A. terreus* on human neutrophils, Naegleria gruberi and Acanthamoeba castellanii. Med. Mycol..

[66] Bertout S., Badoc C., Mallie M., Giaimis J., Bastide J.M. (2002). Spore diffusate isolated from some strains of *Aspergillus fumigatus* inhibits phagocytosis by murine alveolar macrophages. FEMS Immunol. Med. Microbiol..

[67] Geib E., Gressler M., Viediernikova I., Hillmann F., Jacobsen I.D., Nietzsche S., Hertweck C., Brock M. (2016). A Non-canonical Melanin Biosynthesis Pathway Protects *Aspergillus terreus* Conidia from Environmental Stress. Cell Chem. Biol..

[68] Dao A.H., Robinson D.P., Wong S.W. (1983). Frequency of *Entamoeba gingivalis* in human gingival scrapings. Am. J. Clin. Pathol..

[69] Vanessa B., Virginie M., Nathalie Q., Marie-Helene R., Christine I. (2012). *Hartmannella vermiformis* can promote proliferation of Candida spp. in tap-water. Water Res..

[70] Koller B., Schramm C., Siebert S., Triebel J., Deland E., Pfefferkorn A.M., Rickerts V., Thewes S. (2016). *Dictyostelium discoideum* as a Novel Host System to Study the Interaction between Phagocytes and Yeasts. Front. Microbiol..

[71] Levrat P., Pussard M., Steinberg C., Alabouvette C. (1991). Regulation of *Fusarium oxysporum* populations introducied into soils: The amoebal predation hypothesis. FEMS Microbiol. Ecol..

[72] Siddiqui R., Lakhundi S., Khan N.A. (2015). Interactions of *Pseudomonas aeruginosa* and *Corynebacterium* spp. with non-phagocytic brain microvascular endothelial cells and phagocytic *Acanthamoeba castellanii*. Parasitol. Res..

[73] Nunes T.E., Brazil N.T., Fuentefria A.M., Rott M.B. (2016). *Acanthamoeba* and *Fusarium* interactions: A possible problem in keratitis. Acta Trop..

[74] Cateau E., Hechard Y., Fernandes B., Rodier M.H. (2004). Free living amoebae could enhance Fusarium oxysporum growth. Fungal Ecol..

[75] Joseph J., Chaurasia S., Sharma S. (2018). Case Report: Corneal Coinfection with Fungus and Amoeba: Report of Two Patients and Literature Review. Am. J. Trop. Med. Hyg..

[76] Magditch D.A., Liu T.B., Xue C., Idnurm A. (2012). DNA mutations mediate microevolution between host-adapted forms of the pathogenic fungus Cryptococcus neoformans. PLoS Pathog..

[77] Lin J., Idnurm A., Lin X. (2015). Morphology and its underlying genetic regulation impact the interaction between Cryptococcus neoformans and its hosts. Med. Mycol..

[78] Neilson J.B., Ivey M.H., Bulmer G.S. (1978). *Cryptococcus neoformans*: Pseudohyphal forms surviving culture with *Acanthamoeba polyphaga*. Infect. Immun..

[79] Chakraborty S., Old K. (1982). Mycophagous soil amoeba: Interactions with three plant pathogenic fungi. Soil Biol. Biochem..

[80] Chakraborty S., Old K.M. (1986). Ultrastructure and Description of a Fungus-Feeding Amoeba, *Trichamoeba mycophaga* n. sp.(Amoebidae, Amoebea), from Australia. J. Protozool..

[81] Bleuler-Martinez S., Butschi A., Garbani M., Walti M.A., Wohlschlager T., Potthoff E., Sabotiĉ J., Pohleven J., Lüthy P., Hengartner M.O. (2011). A lectin-mediated resistance of higher fungi against predators and parasites. Mol. Ecol..

[82] Corsaro D., Kohsler M., Wylezich C., Venditti D., Walochnik J., Michel R. (2018). New insights from molecular phylogenetics of amoebophagous fungi (Zoopagomycota, Zoopagales). Parasitol. Res..

[83] Vohnik M., Burdikova Z., Albrechtova J., Vosatka M. (2009). Testate amoebae (Arcellinida and Euglyphida) vs. Ericoid mycorrhizal and DSE fungi: A possible novel interaction in the mycorrhizosphere of ericaceous plants?. Microb. Ecol..

[84] De Sousa J.R.P., Goncalves V.N., de Holanda R.A., Santos D.A., Bueloni C., Costa A.O., Petry M.V., Rosa C.A., Rosa L.H. (2017). Pathogenic potential of environmental resident fungi from ornithogenic soils of Antarctica. Fungal Biol..

[85] McClelland E.E., Nicola A.M., Prados-Rosales R., Casadevall A. (2010). Ab binding alters gene expression in Cryptococcus neoformans and directly modulates fungal metabolism. J. Clin. Investig..

[86] Casadevall A., Pirofski L.A. (2007). Accidental virulence, cryptic pathogenesis, martians, lost hosts, and the pathogenicity of environmental microbes. Eukaryot. Cell.

[87] Salah I.B., Ghigo E., Drancourt M. (2009). Free-living amoebae, a training field for macrophage resistance of mycobacteria. Clin. Microbiol. Infect..

[88] Huws S.A., Morley R.J., Jones M.V., Brown M.R., Smith A.W. (2008). Interactions of some common pathogenic bacteria with Acanthamoeba polyphaga. FEMS Microbiol. Lett..

[89] Greub G., Raoult D. (2004). Microorganisms resistant to free-living amoebae. Clin. Microbiol. Rev..

[90] Casadevall A. (2007). The cards of virulence and the global virulome. Microbe.

[91] Araujo Gde S., Fonseca F.L., Pontes B., Torres A., Cordero R.J., Zancope-Oliveira R.M., Casadevall A., Viana N.B., Nimrichter L., Rodrigues M.L. (2012). Capsules from pathogenic and non-pathogenic *Cryptococcus* spp. manifest significant differences in structure and ability to protect against phagocytic cells. PLoS ONE.

